# Supplementary material for: The Eco-Evo Mandala: Simplifying Bacterioplankton Complexity into Ecohealth Signatures
Source: Entropy (Basel). 2021 Nov 8;23(11):1471. doi: 10.3390/e23111471 (PMC8625105; doi:10.3390/e23111471)
Supplement: Supplementary file 1 [file entropy-23-01471-s001.zip › entropy-1379973-supplementary.pdf]

The Eco-Evo Mandala: Simplifying Bacterioplankton Complexity into Ecohealth Signatures

- Supplementary Information -

Elroy Galbraith[a*], **Matteo Convertino**[b]

[a] Graduate School of Information Science and Technology, Hokkaido University, Sapporo 060-0814, Japan

[b] bluEco Lab, Institute of Environment and Ecology, Tsinghua Shenzhen International Graduate School, Tsinghua University, Shenzhen 518055 China

November 6, 2021

Corresponding author: * elroy-louismatthias.galbraith-.d1@elms.hokudai.ac.jp; Tel.: +81-011-706-6489

Keywords: marine microbiome; ecosystem health; biocomplexity; optimality; biogeochemical forcing; climate change; Mandala

Supplementary Figure Captions

Figure S1. Power-law Distribution of Abundance. (A) The EPDF of abundance for populations (colored lines) and the communities (black lines). (B) The top 10 phyla given the exponent of the distribution of their abundance.

Figure S2. Taylor's Law of Abundance. (A) The relation between the variance of OTU abundance and the mean of OTU abundance for populations (colored points) and the communities (black lines). (B) The top 10 phyla given the Taylor's law exponent of their abundance.

Figure S3. Exponential Distribution of Interaction (A) The EPDF of interactions (TE) for populations (colored lines) and the communities (black lines). (B) The top 10 Phyla given the exponent of the distribution of their interactions.

Figure S4. Phylogenetic Dissimilarity (A) The phylogenetic trees inferred for each site given the taxonomic classification of all OTUs observed. A colored node and edge indicates that the taxon was observed in that site; a grey node or edge was not observed. (B) The top 10 Phyla given their phylogenetic distances.

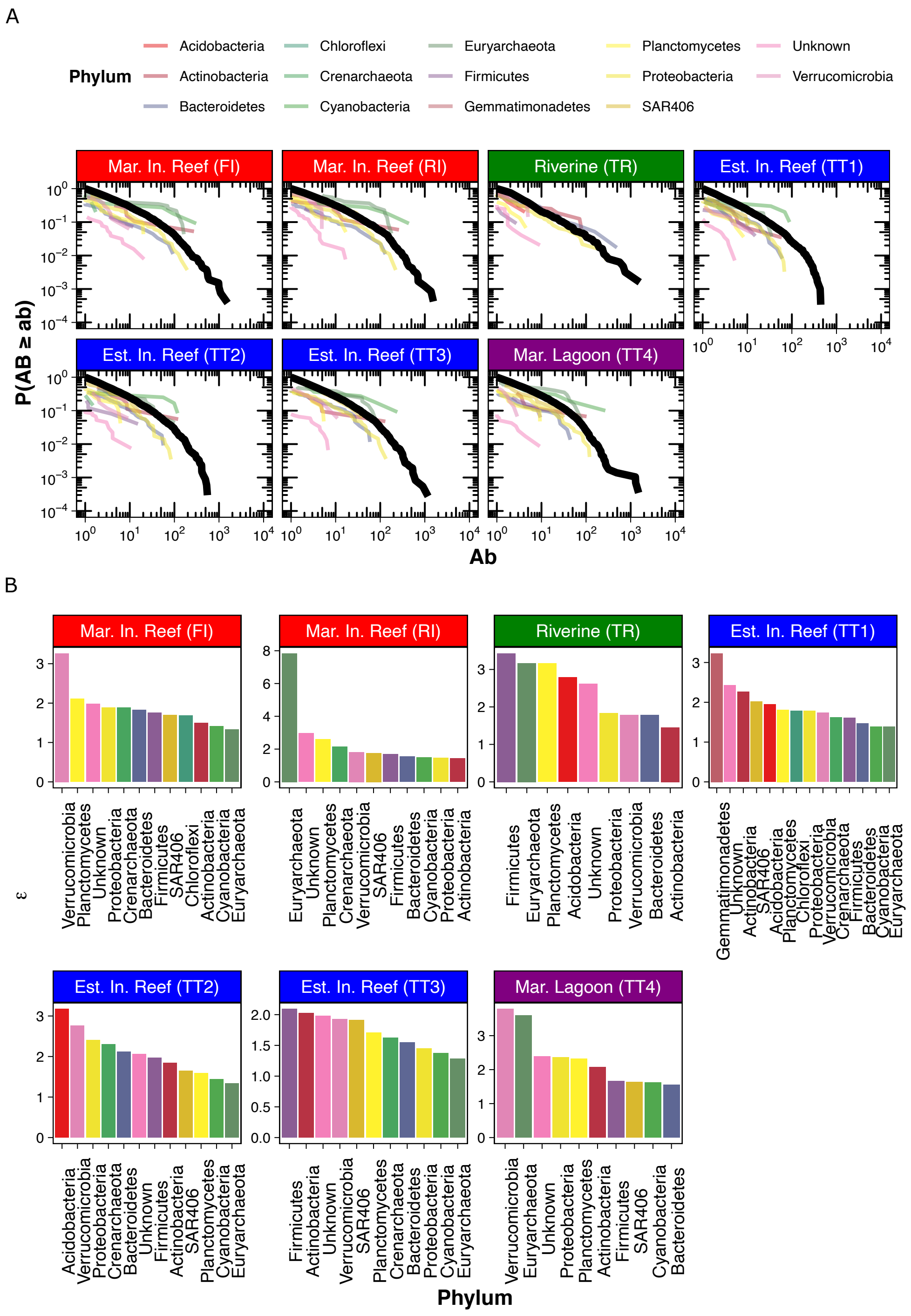
A
Phylum
Acidobacteria
Actinobacteria
Bacteroidetes
Chloroflexi
Crenarchaeota
Cyanobacteria
Euryarchaeota
Firmicutes
Gemmatimonadetes
Planctomycetes
Proteobacteria
SAR406
Unknown
Verrucomicrobia
Mar. In. Reef (FI)
Mar. In. Reef (RI)
Riverine (TR)
Est. In. Reef (TT1)
Est. In. Reef (TT2)
Est. In. Reef (TT3)
Mar. Lagoon (TT4)
P(AB ≥ ab)
Ab
B
ε
Phylum

Figure S1:

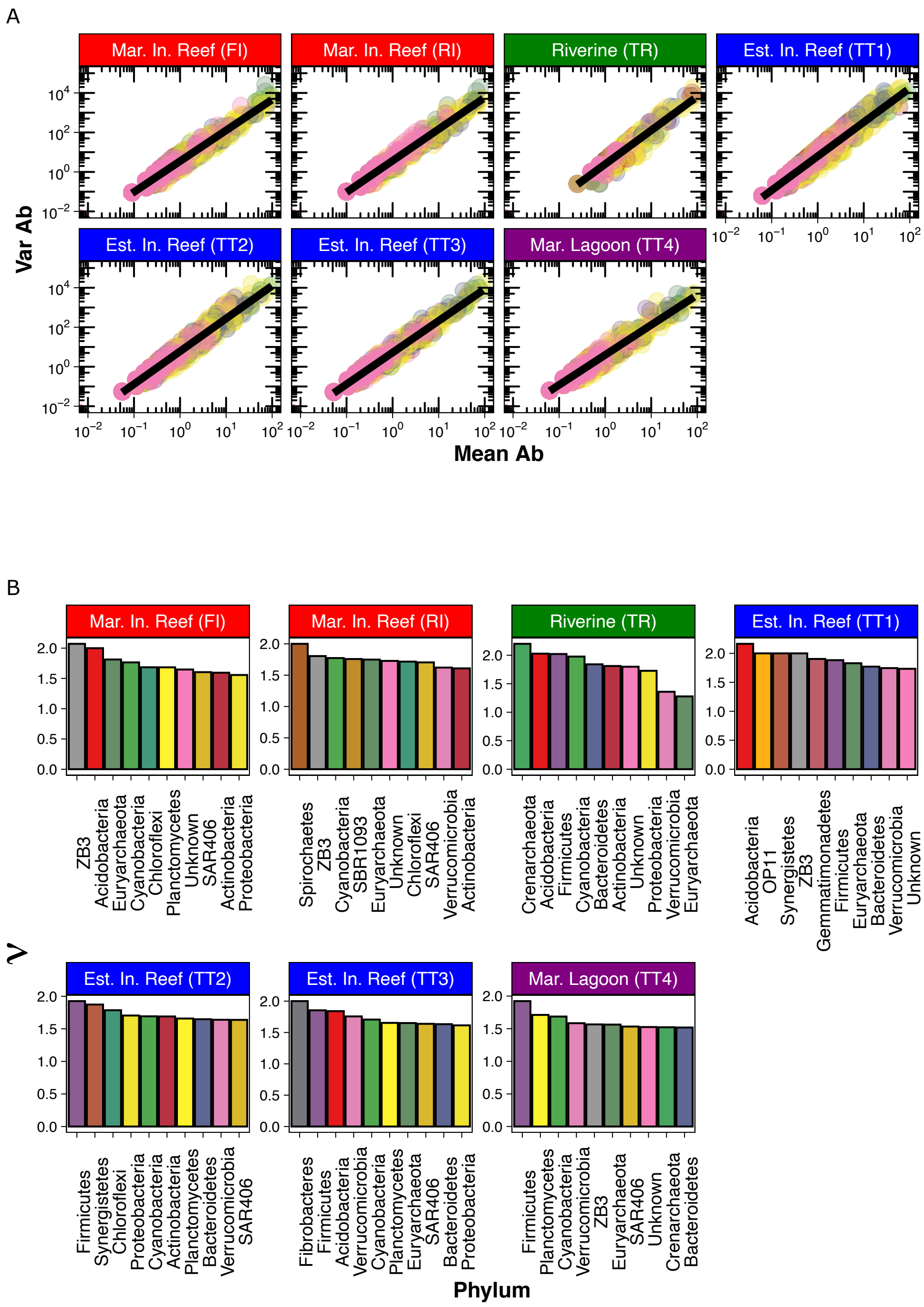
A
Mar. In. Reef (FI)
Mar. In. Reef (RI)
Riverine (TR)
Est. In. Reef (TT1)
Est. In. Reef (TT2)
Est. In. Reef (TT3)
Mar. Lagoon (TT4)
Var Ab
Mean Ab
B
ZB3
Acidobacteria
Euryarchaeota
Cyanobacteria
Chloroflexi
Planctomycetes
Unknown
SAR406
Actinobacteria
Proteobacteria
Spirochaetes
SBR1093
Verrucomicrobia
Crenarchaeota
Firmicutes
Bacteroidetes
OP11
Synergistetes
Gemmatimonadetes
Fibrobacteres
ν
Phylum

Figure S2:

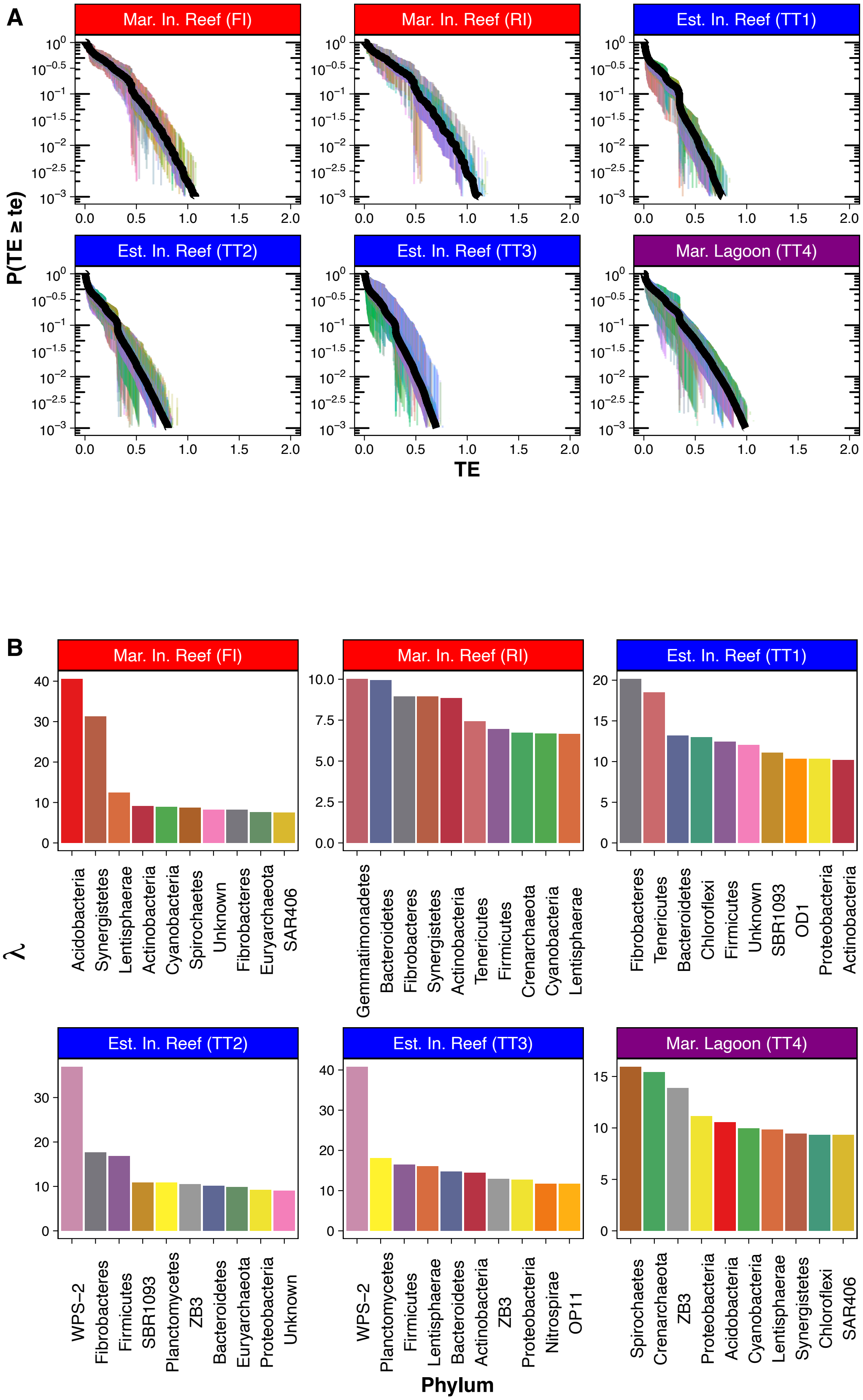
A
Mar. In. Reef (FI)
Mar. In. Reef (RI)
Est. In. Reef (TT1)
Est. In. Reef (TT2)
Est. In. Reef (TT3)
Mar. Lagoon (TT4)
P(TE ≥ te)
TE
B
Mar. In. Reef (FI)
Acidobacteria Synergistetes Lentisphaerae Actinobacteria Cyanobacteria Spirochaetes Unknown Fibrobacteres Euryarchaeota SAR406
Mar. In. Reef (RI)
Gemmatimonadetes Bacteroidetes Fibrobacteres Synergistetes Actinobacteria Tenericutes Firmicutes Crenarchaeota Cyanobacteria Lentisphaerae
Est. In. Reef (TT1)
Fibrobacteres Tenericutes Bacteroidetes Chloroflexi Firmicutes Unknown SBR1093 OD1 Proteobacteria Actinobacteria
Est. In. Reef (TT2)
WPS−2 Fibrobacteres Firmicutes SBR1093 Planctomycetes ZB3 Bacteroidetes Euryarchaeota Proteobacteria Unknown
Est. In. Reef (TT3)
WPS−2 Planctomycetes Firmicutes Lentisphaerae Bacteroidetes Actinobacteria ZB3 Proteobacteria Nitrospirae OP11
Mar. Lagoon (TT4)
Spirochaetes Crenarchaeota ZB3 Proteobacteria Acidobacteria Cyanobacteria Lentisphaerae Synergistetes Chloroflexi SAR406
λ
Phylum

Figure S3:

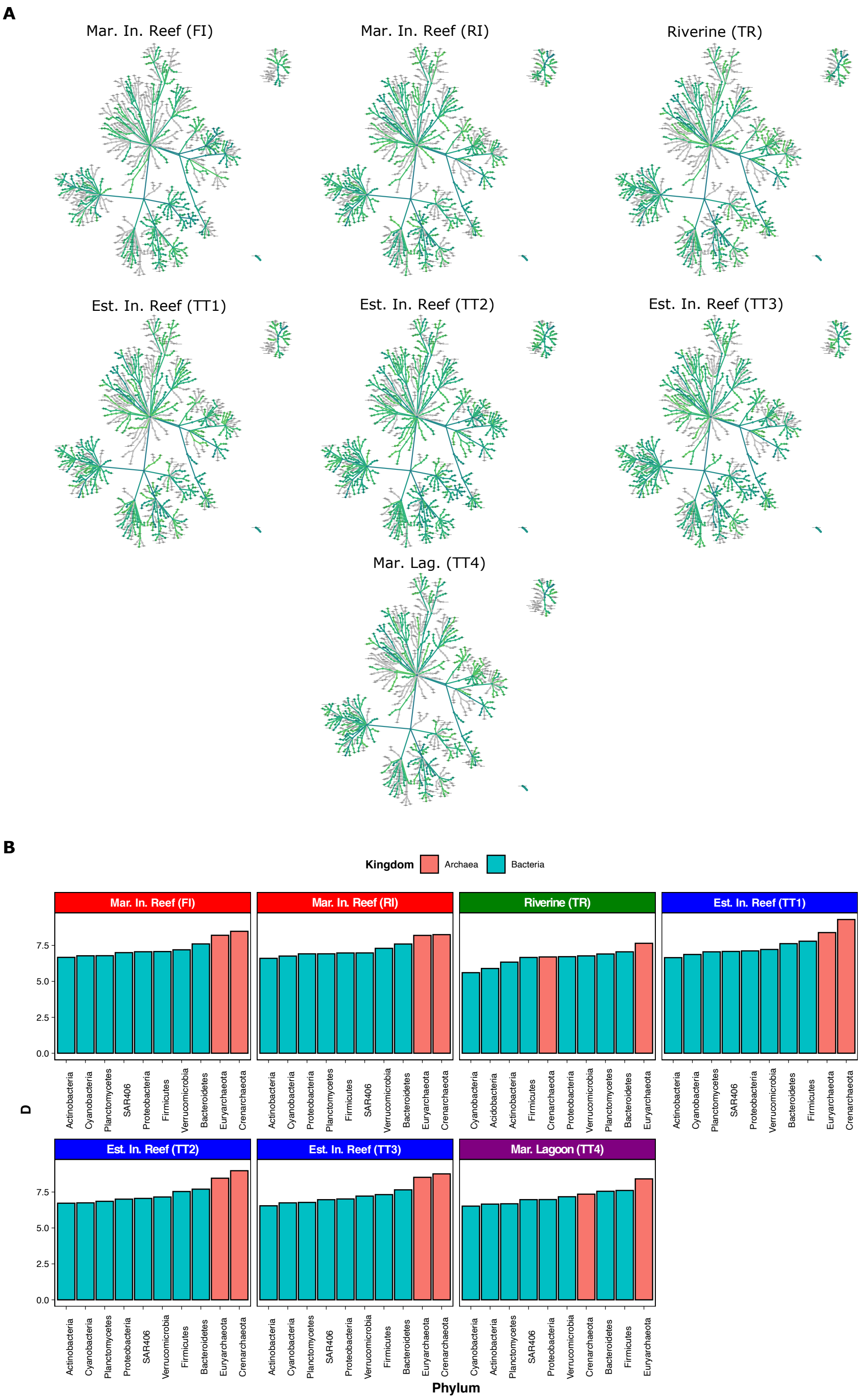
A
Mar. In. Reef (FI)
Mar. In. Reef (RI)
Riverine (TR)
Est. In. Reef (TT1)
Est. In. Reef (TT2)
Est. In. Reef (TT3)
Mar. Lag. (TT4)
B
Kingdom
Archaea
Bacteria
Mar. In. Reef (FI)
Mar. In. Reef (RI)
Riverine (TR)
Est. In. Reef (TT1)
Est. In. Reef (TT2)
Est. In. Reef (TT3)
Mar. Lagoon (TT4)
0.0
2.5
5.0
7.5
D
Actinobacteria
Cyanobacteria
Planctomycetes
SAR406
Proteobacteria
Firmicutes
Verrucomicrobia
Bacteroidetes
Euryarchaeota
Crenarchaeota
Acidobacteria
Phylum

Figure S4:
